# Supplementary material for: Wood structure explained by complex spatial source-sink interactions
Source: Nat Commun. 2022 Dec 19;13:7824. doi: 10.1038/s41467-022-35451-7 (PMC9763502; doi:10.1038/s41467-022-35451-7)
Supplement: Supplementary file 1 — Supplementary information [file 41467_2022_35451_MOESM1_ESM.pdf]

Supplementary Figures

For the publication: Wood structure explained by complex spatial source-sink interactions

Authors: Andrew D. Friend, Annemarie H. Eckes-Shephard, Quinten Tupker

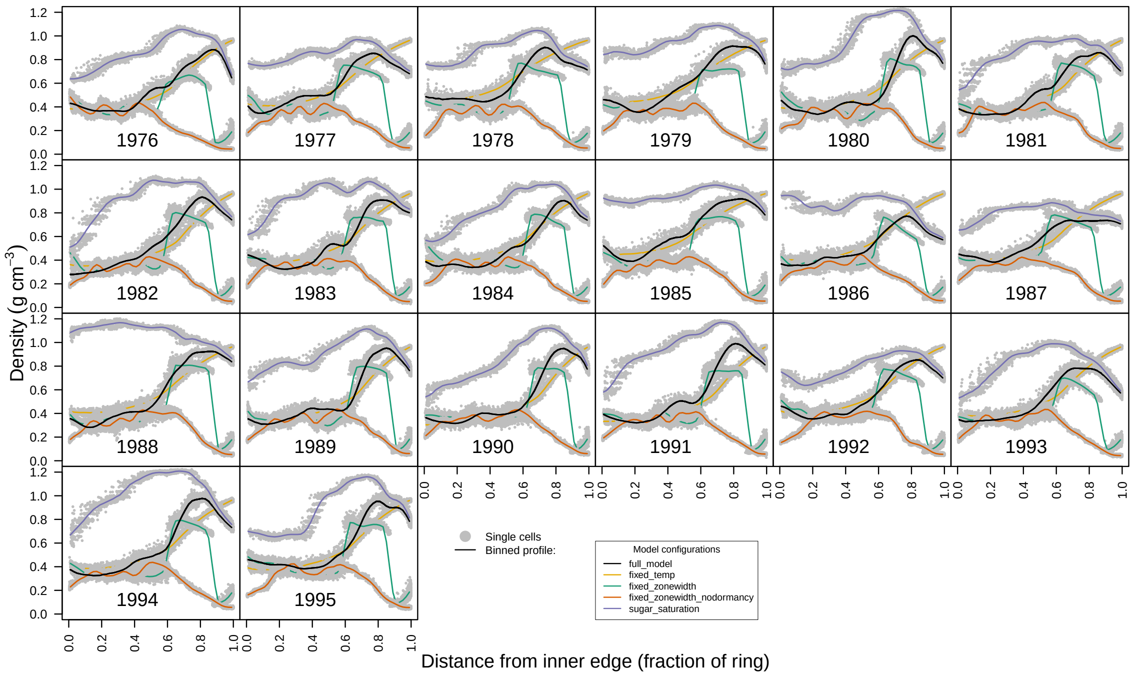

Supplementary Figure 1. Annual standardised wood-density profiles for individual model configuration runs (cf. Fig. 2, main text) over 1972–1995. Solid lines were derived from simulated cell-mass density profiles (grey dots), standardised across 100 independent radial files per simulation ("Model configurations") using the binning method described in the caption of Fig. 2, main text.

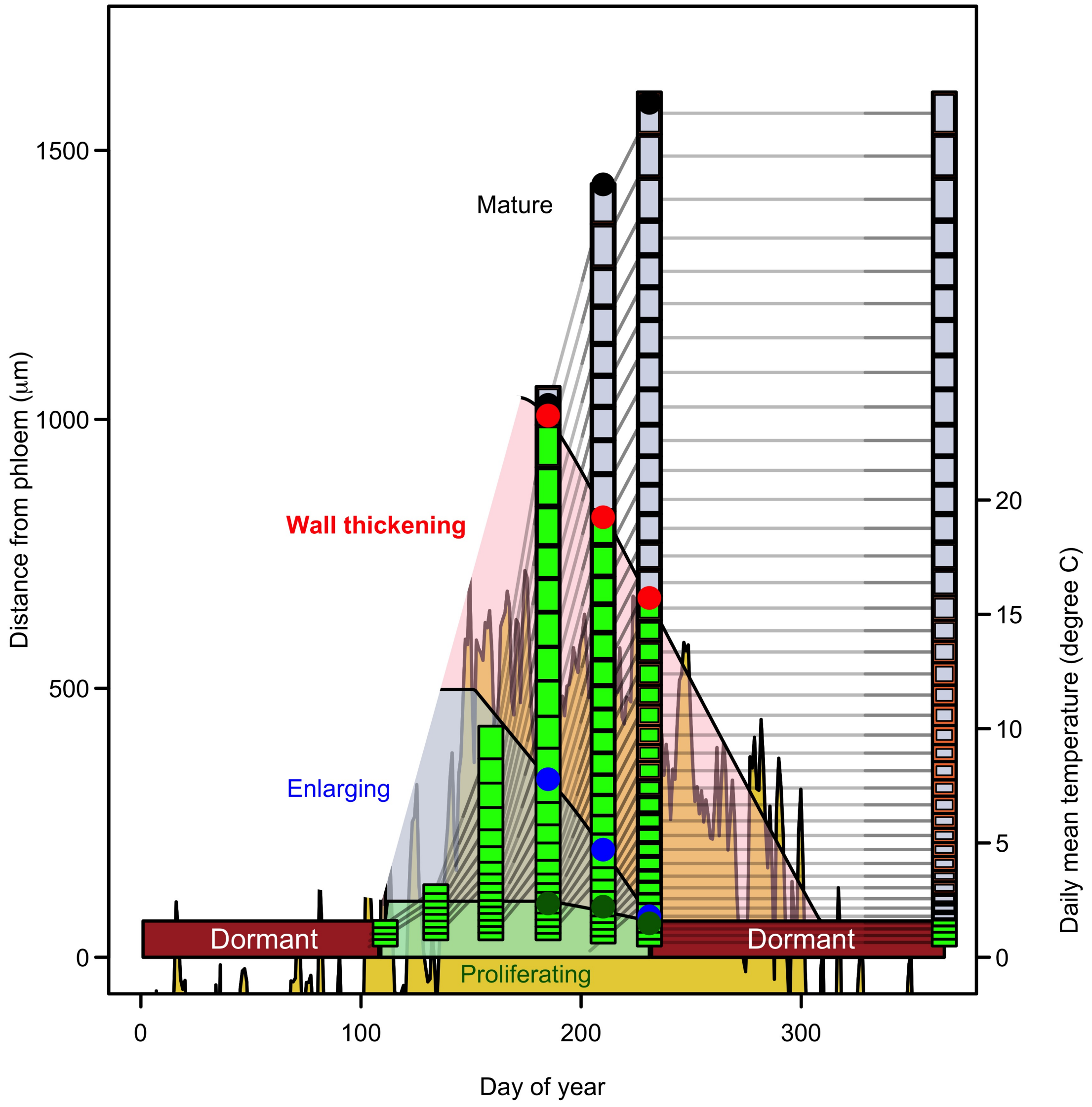

Supplementary Figure 2. Simulated radial file development from the full-model simulation in Fig 2 in the main text. Cells are means over 100 radial files. Cells positions are shown relative to the phloem, and are therefore represented as moving through development zones as they undergo differentiation. Filled circles are observed developmental zone boundaries (dark green: proliferating, blue: enlarging-only, and red: wall thickening) and total file length (black)[23] (see "Observations"). Dormancy affects enlargement in both proliferation and enlarging cells. Living cells are shown with green protoplasm and mature, dead cells with grey lumen. Cell walls are orange and are drawn to the left y-axis scale. The influence of temperature (yellow zone in background) on wall thickness is limited when the proliferation and enlargement-only zones are active due to the wall-thickening zone rapidly retreating and the large distance from the phloem, reducing carbohydrate concentrations. In contrast, wall thickening in latewood cells occurs closer to the phloem and for longer, leading to higher densities and strong effects of temperature.

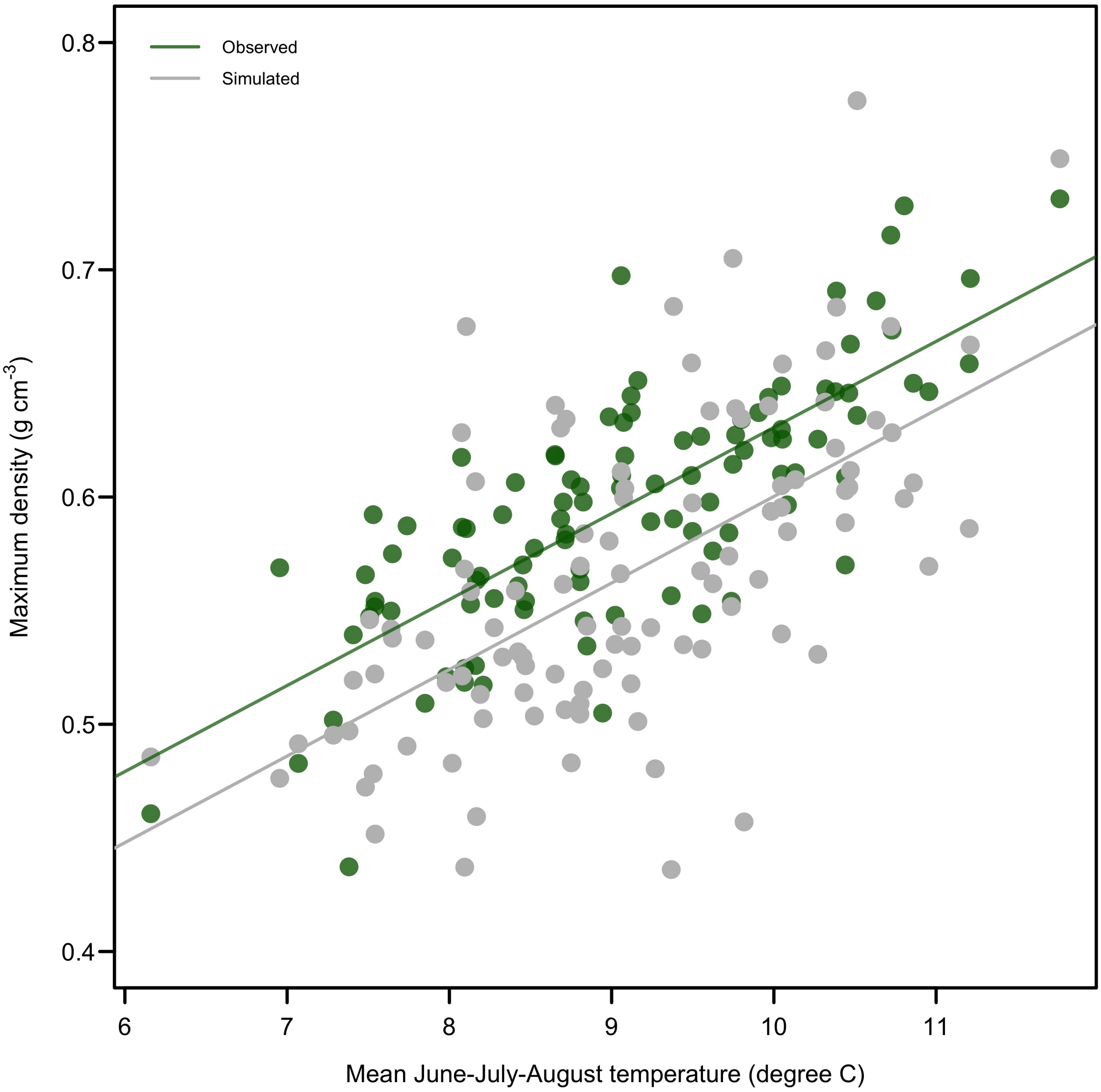

Supplementary Figure 3. Observed and simulated relationships between annual maximum ring density and mean June-July-August temperature in northern Sweden. Simulation as for the full model in Fig. 2 in the main text, except over 1901-2004, with latitude = 68.26°N, and temperatures from the relevant half-degree grid-point in a global dataset[43]. Observations are "Tree Ring Maximum Density Chronology, Regional Curve Standardization" from ftp://ftp.ncdc.noaa.gov/pub/data/paleo/treering/reconstructions/europe/sweden/tornetrask-temperature2008.txt[31], scaled using the observed mean of 0.615 g cm^{-3} [31]. Model parameter E_{aw} was adjusted to achieve the same slope as observed (Table 1, main text), which means that absolute densities were not calibrated.

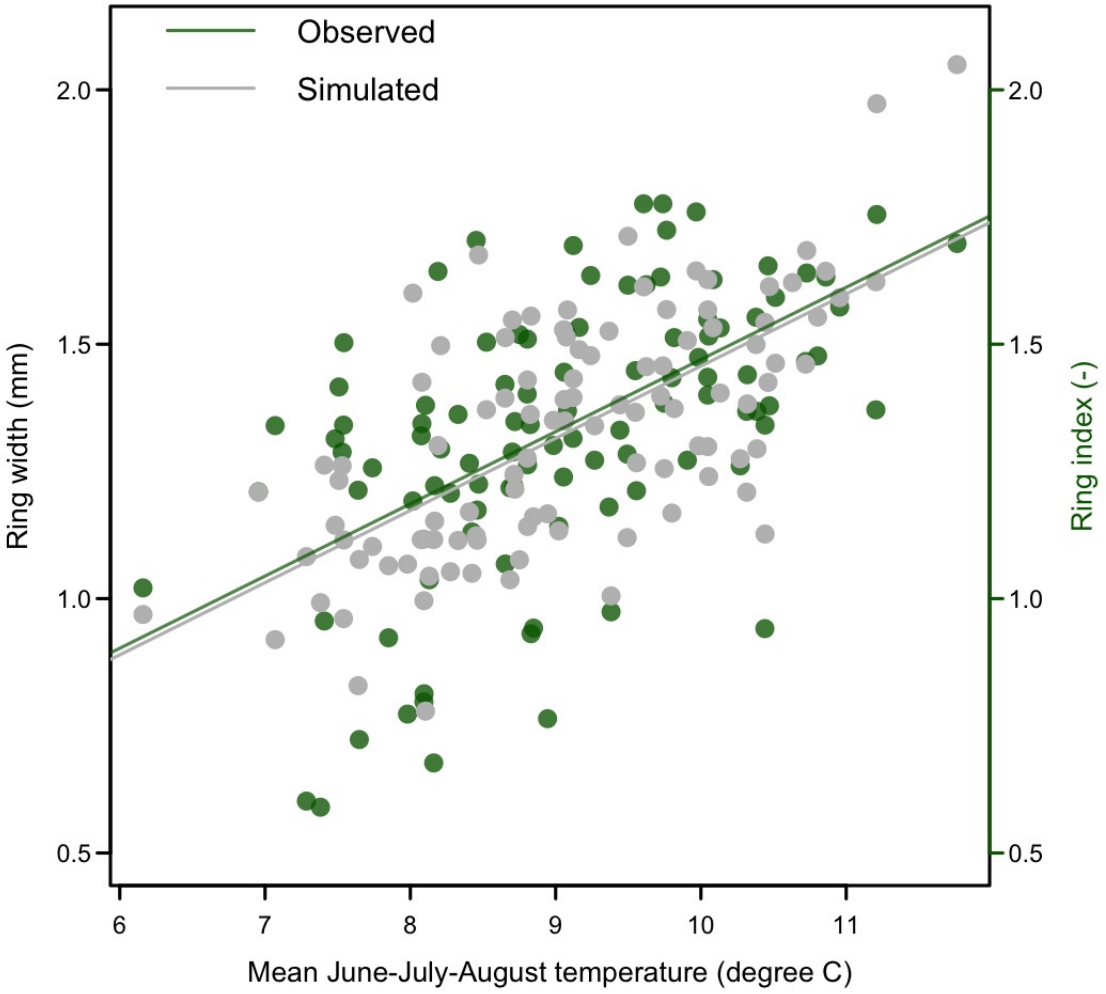

Supplementary Figure 4. Observed and simulated relationships between annual ring width and mean June-July-August temperature in northern Sweden. Simulation undertaken as in Fig. 3, main text. Observations are "Tree Ring Width Chronology, Regional Curve Standardization" from ftp://ftp.ncdc.noaa.gov/pub/data/paleo/treering/reconstructions/europe/sweden/tornetrask-temperature2008.txt[31]. Model parameter E_a was adjusted to achieve the same slope as observed (Table 1, main text), which means that absolute widths (mm) were not calibrated to the observations (Ring index).

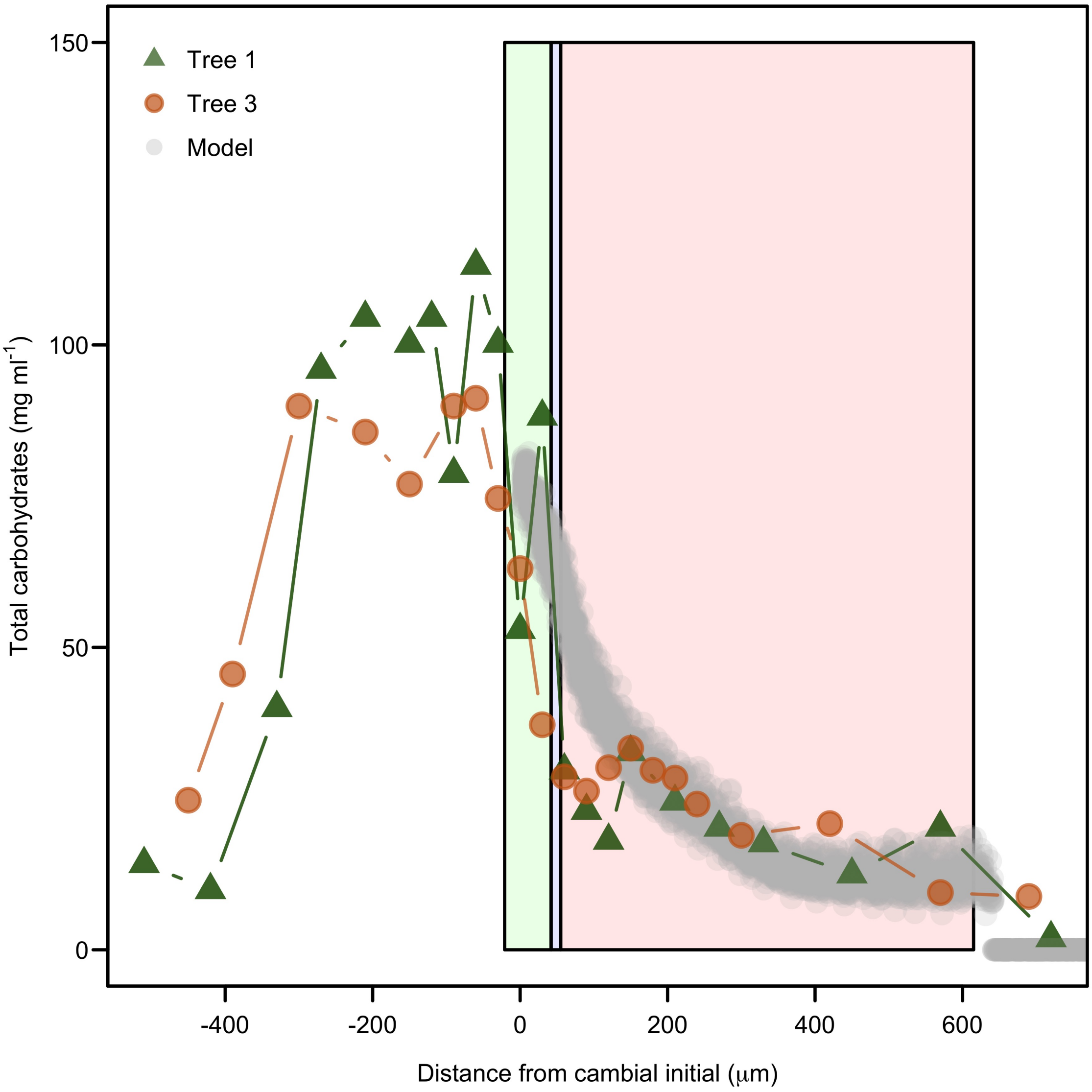

Supplementary Figure 5. Observed[23] and simulated distributions of carbohydrates across the developing wood on DOY 231. Model results are values for individual cells across 100 files, as for the full-model simulation in Fig. 2, main text. Shaded light green area is the observed proliferation zone, shaded light blue area is the observed enlargement-only zone, and shaded light red area is the observed wall-thickening zone.
